# Improving the Accuracy of Length of stay Risk Adjustment Models using Linked Data

**DOI:** 10.23889/ijpds.v1i1.382

**Published:** 2017-04-19

**Authors:** Tolulope Sajobi, Mingshan Lu, Jason Jiang, Hude Quan

**Affiliations:** 1 University of Calgary

## Background

Hospital length of stay (LOS) is a widely used measure for assessing cross-jurisdiction health system performance and informs resource allocation decisions. However, the accuracy of existing LOS risk adjustment models are limited, because they are mostly derived from administrative data, which mostly contain clinical/diagnostic information but lack detailed information on relevant demographic, socio-economic (SES), and self-reported health-related quality of life (HRQOL) risk factors, which have been shown to improve the accuracy of LOS risk adjustment models. The study investigates the relative contribution of demographic, socio-economic, and health status risk factors derived through data linkage in improving the accuracy of LOS risk adjustment models. 

## Methods

Population-based data on 8000 individuals hospitalized for coronary heart disease were obtained from Alberta Provincial Project on Outcomes Assessment in Coronary Heart Disease (APPROACH) registry and linked to Alberta Discharge Abstract Database (DAD). SES was measured using multi-domain measure of SES derived from area-level census information, while the health-related quality of life outcome was measured using the Seattle Angina Questionnaire. LOS risk adjustment model based on hierarchical logistic regression models was developed to assess relative impact of each SES measure and HRQOL measure improving the predictive accuracy of LOS adjustment models. The relative impact of each predictor was assessed by its adjusted odds ratio (OR) and improvement over the predictive accuracy of a reference model that included patients' clinical risk factors only. 

## Result

More than 80% of the hospitalized individuals had prolonged LOS more than 10 days. The HRQOL and single-domain measures of SES had significant impact in accurately predicting LOS. But the inclusion of the multi-domain measure SES did not significantly improve the accuracy of LOS risk adjustment models

## Conclusions

Using large population-based Canadian data, our study suggests that the inclusion of patients' SES and health status information through data linkage can improve the accuracy of LOS risk adjustment models. The development of more accurate risk adjustment models can aid the identification of individuals at risk of prolonged LOS and comparison of health system performance across several cross-jurisdictions.

